# Risk factors for subsequent vertebral fracture after percutaneous vertebroplasty in patients with osteoporotic vertebral compression fractures in Northern China

**DOI:** 10.3389/fsurg.2026.1786765

**Published:** 2026-03-10

**Authors:** Yafeng Guo, Yaheng Zhao, Qi Sun, Hongyang Gao, Wei Zhang

**Affiliations:** 1 The First Hospital of Hebei Medical University, Shijiazhuang, China; 2 The Third Hospital of Hebei Medical University, Shijiazhuang, China

**Keywords:** osteoporosis, osteoporotic vertebral compression fracture, percutaneous vertebroplasty, risk factor, subsequent vertebral fracture

## Abstract

**Background:**

Osteoporotic vertebral compression fractures (OVCF) are commonly treated with percutaneous vertebroplasty (PVP). However, Subsequent vertebral fracture (SVF) following PVP surgery, as a serious complication, has attracted widespread attention. The purpose of this study was to investigate the risk factors for SVF after PVP in elderly patients with OVCF.

**Methods:**

This study retrospectively analyzed elderly patients who underwent PVP for single-level OVCF. Patients were divided into the SVF group and the control group. Demographic, surgery-related, and imaging parameters were collected. Independent risk factors were identified through univariate and multivariate logistic regression analyses.

**Results:**

A total of 162 elderly patients over 60 years old with single-level OVCF treated by PVP were included in this study, among whom 27 patients developed SVF. Logistic regression analysis revealed that low body mass index (BMI) (OR = 0.608; 95% CI = 0.426–0.867, *P* = 0.006), intradiscal cement leakage (OR = 10.993; 95% CI = 2.391–50.540, *P* = 0.002), and low Hounsfield unit (HU) values (OR = 0.958; 95% CI = 0.923–0.995, *P* = 0.025) were significantly associated with SVF after PVP for the treatment of OVCF and were identified as independent risk factors.

**Conclusion:**

Low BMI, intradiscal cement leakage, and low HU values were independent risk factors for SVF after PVP surgery for the treatment of OVCF.

## Introduction

Osteoporosis is recognized as a common chronic musculoskeletal disease, characterized by reduced bone mass, decreased bone density, and increased risk of fractures (1). Among individuals aged 60 and above, osteoporotic vertebral compression fracture (OVCF) is one of the most common fracture types secondary to osteoporosis (2). China has nearly 100 million osteoporosis patients, with the North China region being particularly affected. As the population ages, the situation is becoming increasingly severe, posing a major public health concern. OVCF causes severe low back pain, limitations in daily activities, spinal kyphosis, reduced quality of life, and can increase the risk of comorbidities and even mortality (3–5). OVCF imposes a significant economic burden and pressure on patients' families and the healthcare system.

Clinically, OVCF is typically managed conservatively. Common conservative treatments include analgesics, bed rest, physical therapy, and bracing. If conservative treatment fails, surgical intervention is recommended for OVCF. Currently, the most widely used minimally invasive surgical procedure is percutaneous vertebroplasty (PVP). PVP offers advantages such as minimal trauma, short operative time, reduced blood loss, rapid recovery, and has demonstrated good safety and therapeutic efficacy (6–8). As a result, PVP is extensively used in the treatment of OVCF. Despite the widespread use of PVP for OVCF, the occurrence of subsequent vertebral fractures (SVF) after the procedure has emerged as a significant concern, emerging as an urgent issue to address (9–12). SVF not only necessitates additional interventions but also exacerbates spinal deformity, functional decline, and healthcare costs, underscoring the urgent need to identify modifiable risk factors.

Currently, there is limited research on SVF after PVP in the patients aged 60 and above with OVCF. A systematic investigation into SVF holds significant clinical and societal value. Therefore, the objective of this study is to explore the risk factors for SVF following PVP in elderly patients with OVCF and to identify corresponding preventive measures. This will not only provide evidence-based clinical guidance for spinal surgeons but may also significantly reduce or prevent the occurrence of SVF after PVP in OVCF patients, thereby alleviating the economic burden on families and conserving valuable medical resources.

## Methods

### Study population

Patients with single-level OVCF who underwent PVP between May 2020 and December 2023 at our hospital were included in this study. Based on the occurrence of SVF, patients were assigned to either SVF group or the control group. The inclusion criteria were as follows: (1) Patients aged 60 years or older; (2) Low back pain caused by low-energy fractures; (3) Single-level OVCF; (4) Initial surgery being PVP; (5) Complete medical records; (6) A follow-up period of at least 12 months. The exclusion criteria were as follows: (1) Patients younger than 60 years; (2) Vertebral fractures caused by high-energy trauma; (3) Multi-level OVCF treated with PVP; (4) Pathological fractures; (5) History of spinal internal fixation surgery; (6) Follow-up period less than 12 months or loss to follow-up. This study was approved and supported by the Ethics Committee of the Third Hospital of Hebei Medical University and complies with the Helsinki Declaration. All patients' informed consent was obtained prior to their inclusion in this study.

### Research indicators

The parameters or indicators included in this study were as follows. Demographic indicators included age, gender, body mass index (BMI), hypertension, diabetes, smoking, alcohol consumption, bisphosphonate use, history of previous vertebral fracture, fracture level, and follow-up duration. Surgery-related parameters included cement volume, cement leakage, and intradiscal cement leakage. Imaging indicators included the preoperative wedge angle, postoperative wedge angle, and Hounsfield unit (HU) value. The wedge angle was defined as the angle formed between the superior and inferior endplates of the fractured vertebral body on Preoperative and postoperative lateral radiographs, measured using the method as described by Sadiqi (13). The HU value was considered a parameter for assessing BMD and was measured from the region of interest (ROI) within the cancellous bone on axial CT images at the L1 vertebrae (14). The ROI was placed as the largest possible oval within the vertebral body of L1, carefully avoiding the cortical bone. All imaging measurements were performed by two experienced physicians.

### Statistical analysis

All data in this study were statistically analyzed using SPSS version 23. Continuous variables conforming to a normal distribution are expressed as mean ± standard deviation. Categorical variables are presented as frequencies. In univariate analysis, continuous variables were analyzed using independent samples *t-*tests or non-parametric tests. Categorical variables were analyzed using the chi-square test or Fisher's exact test. Variables with a *p*-value < 0.05 were included in the multivariate logistic regression analysis. Multivariate logistic regression was employed to identify independent risk factors. A *p*-value < 0.05 was considered statistically significant.

## Results

A total of 162 patients who underwent PVP for the treatment of OVCF were included in this study, comprising 36 males and 126 females, with a mean age and BMI of 73.44 ± 7.64 years and 22.25 ± 2.09 kg/m², respectively. Among these 162 patients, 27 developed vertebral refracture after PVP, while the remaining 135 did not. In the SVF group, the 27 patients included 7 males and 20 females, with a mean age and BMI of 77.22 ± 7.15 years and 20.94 ± 1.36 kg/m², respectively (Table 1).

**Table 1:** Baseline characteristics and univariate analysis of variables in the SVF and control groups.

Variable	All patients	Control group	SVF group	*P*
	(*n* = 162)	(*n* = 135)	(*n* = 27)	
Age (years)	73.44 ± 7.64	72.69 ± 7.54	77.22 ± 7.15	0.005
Gender				0.612
Male	36	29	7	
Female	126	106	20	
BMI (kg/m^2^)	22.25 ± 2.09	22.51 ± 2.11	20.94 ± 1.36	<0.001
Hypertension, *n* (%)				0.343
No	103	88	15	
Yes	59	47	12	
Diabetes, *n* (%)				0.357
No	125	106	19	
Yes	37	29	8	
Smoking, *n* (%)				0.724
No	130	109	21	
Yes	32	26	6	
Alcohol consumption, *n* (%)				0.527
No	143	120	23	
Yes	19	15	4	
Bisphosphonate use				0.214
No	103	83	20	
Yes	59	52	7	
Previous vertebral history				0.048
No	142	122	20	
Yes	20	13	7	
Fracture level, *n* (%)				0.106
Non-TL junction	77	68	9	
TL junction	85	67	18	
Cement volume, mL	4.37 ± 0.83	4.36 ± 0.85	4.41 ± 0.71	0.800
Cement leakage				0.407
No	124	105	19	
Yes	38	30	8	
Intradiscal cement leakage				0.030
No	146	125	21	
Yes	16	10	6	
Preoperative wedge angle,°	12.27 ± 7.14	12.14 ± 6.52	12.94 ± 9.80	0.594
Postoperative wedge angle,°	8.63 ± 6.11	8.18 ± 5.34	10.88 ± 8.81	0.036
HU value	76.76 ± 26.90	80.31 ± 26.65	58.98 ± 20.60	<0.001
Follow-up duration, months	14.43 ± 1.76	14.46 ± 1.90	14.30 ± 0.78	0.662

SVF, subsequent vertebral fracture; BMI, body mass index; PVP, percutaneous vertebroplasty; TL, thoracolumbar; HU, Hounsfield unit.

### Univariate analysis identified the significant factors

In the univariate logistic regression analysis, the age of patients in the SVF group was significantly older than that in the control group (*P* < 0.05), while the BMI was significantly lower (*P* < 0.05). Additionally, patients with a history of previous vertebral fracture exhibited a significantly higher risk of SVF (*P* < 0.05). Statistically significant differences were also observed between the two groups in terms of intradiscal cement leakage, postoperative wedge angle, and HU values (*P* < 0.05) (Table 1).

### Multivariate logistic regression model identified the independent risk factors

In the multivariate regression analysis, after adjusting for confounding factors, BMI (OR = 0.608; 95% CI = 0.426–0.867, *P* = 0.006), intradiscal cement leakage (OR = 10.993; 95% CI = 2.391–50.540, *P* = 0.002), and HU values (OR = 0.958; 95% CI = 0.923–0.995, *P* = 0.025) were identified as independent risk factors for SVF after PVP in OVCF patients. Among these, intradiscal cement leakage was significantly positively correlated with vertebral refracture after PVP in OVCF patients, while BMI and HU values were significantly negatively correlated with it (Table 2).

**Table 2:** Multivariate regression analysis of factors associated with SVF.

Variable	B	SE	Wald	OR (95% CI)	*P*
Age, years	−0.061	0.066	0.875	0.940 (0.827–1.070)	0.350
BMI, kg/m^2^	−0.498	0.182	7.530	0.608 (0.426–0.867)	0.006
Previous vertebral history, *n* (%)	1.033	0.614	2.832	2.808 (0.844–9.349)	0.092
Intradiscal cement leakage, *n* (%)	2.397	0.778	9.487	10.993 (2.391–50.540)	0.002
Postoperative wedge angle,°	0.040	0.039	1.012	1.040 (0.963–1.124)	0.314
HU value	−0.042	0.019	5.003	0.958 (0.923–0.995)	0.025

SVF, subsequent vertebral fracture; BMI, body mass index; HU, Hounsfield unit; B, unstandardized B coefficient; S.E., standard error; OR, odds ratio; CI, confidence interval.

## Discussion

With the intensification of aging, the number of patients with osteoporotic fractures is increasing. PVP is widely used in the treatment of OVCF; however, postoperative new vertebral fractures have attracted widespread attention. In our study, a total of 27 patients experienced SVF after undergoing PVP for the treatment of OVCF, accounting for 16.67% of the cases. The results of this study indicate that previous vertebral history is positively independent risk factor, while BMI and HU values are significantly negatively correlated with SVF and are identified as protective factors. The negative correlation indicates that higher BMI and higher HU values are associated with a lower risk of SVF, suggesting that these factors may serve as protective factors. In other words, as BMI or HU values increase, the probability of postoperative vertebral refracture decreases.

The results of this study indicate a significant negative correlation between BMI and SVF after PVP in OVCF patients, meaning that a low BMI significantly increases the risk of refracture. This finding is consistent with previous research. Fang et al. (15) conducted a retrospective analysis of 445 elderly patients and found that patients with lower BMI had a higher risk of SVF. In a retrospective study involving 371 patients, Bian et al. (16) explored the risk factors for new vertebral fractures after percutaneous vertebral augmentation in the elderly, concluding that BMI was not significantly correlated with postoperative vertebral refracture. However, in another retrospective study, Li et al. (17) analyzed 385 patients and identified BMI as a risk factor for new vertebral fractures after PVP in osteoporotic fracture patients, with a high BMI significantly increasing the risk of SVF. We believe that, in the elderly, a low BMI likely reflects poorer overall physical condition. Osteoporotic fracture patients should improve their nutritional status and increase their BMI to reduce the risk of SVF after PVP.

According to the literature, whether a history of previous vertebral fracture is a risk factor for vertebral refracture after PVP for OVCF remains controversial. In a retrospective study involving 109 patients, Chen et al. (18) explored various risk factors for SVF after percutaneous vertebral augmentation; however, previous vertebral fracture history was not identified as an independent risk factor. Nevertheless, the sample size of that study was relatively small, and its conclusions require validation through larger-scale studies. In another retrospective study, Cheng et al. (19) analyzed 682 patients to investigate risk factors for SVF after vertebral augmentation and concluded that previous vertebral fracture history significantly increases the risk of refracture. Previous vertebral fractures may alter spinal sagittal alignment and load distribution, thereby increasing mechanical stress on adjacent segments. Briggs et al. (20) demonstrated that vertebral wedge deformities modify the lever arms and increase flexion moments, predisposing to further fractures. These biomechanical alterations may explain the elevated refracture risk in patients with prior vertebral fractures. However, our findings are inconsistent with those of previous studies (21). Based on the findings of this study, previous vertebral fracture history was not an independent risk factor for SVF after PVP in OVCF patients.

According to the findings of this study, intradiscal cement leakage is an independent risk factor for SVF after PVP in OVCF patients and can significantly increase the risk of postoperative refracture. In a retrospective cohort study, Lu et al. (22) analyzed 240 patients and found that intradiscal cement leakage was not a risk factor for vertebral refracture after PVP for the treatment of OVCF. Conversely, in a study involving 421 patients, Zhong et al. (23) investigated the risk factors for vertebral refracture after PVP for OVCF and developed a predictive model. Their results indicated that intradiscal cement leakage was identified as an independent risk factor. Similarly, in a meta-analysis that included 16 studies involving 2,549 patients, Zhang et al. (24) demonstrated that intradiscal cement leakage is a risk factor for adjacent segment fractures after percutaneous vertebral augmentation. These findings are consistent with the results of our study. We consider that intradiscal leakage may accelerate intervertebral disc degeneration and alter local biomechanics, thereby increasing the risk of refracture. Clinicians should strive to minimize and avoid disc leakage during surgical procedures to reduce the occurrence of refracture.

Our study demonstrates that HU values exhibit a significant negative correlation with refracture after PVP in OVCF patients and are identified as an independent risk factor for postoperative vertebral refracture. HU values measured by CT are widely used to assess bone mineral density. Compared with the classical T-score determined by dual-energy x-ray absorptiometry, HU values offer unique advantages, particularly in cases of scoliosis or degenerative spinal vertebrae. In a study involving 349 postmenopausal women who underwent percutaneous vertebral augmentation, Chang et al. (25) concluded that low HU values are an independent predictor of postoperative vertebral refracture. In a retrospective study of 247 patients, Cheng et al. (26) found that low HU values were an independent risk factor for vertebral refracture after PVP or PKP treatment for OVCF. Furthermore, in a systematic review and meta-analysis comprising 18 studies and involving 7,743 patients, Liu et al. (27) investigated the risk factors for vertebral refracture after percutaneous vertebral augmentation for OVCF. Their results indicated that HU values were significantly associated with postoperative vertebral refracture and served as an independent risk factor. We believe that low HU values reflect more severe osteoporosis and higher bone fragility, thereby increasing the risk and incidence of postoperative vertebral refracture. OVCF patients after PVP should receive systematic anti-osteoporosis treatment to improve bone mineral density and reduce the occurrence of postoperative vertebral refracture.

The identification of low BMI, intradiscal cement leakage, and low HU values as independent risk factors for SVF offers actionable insights for clinical practice. Preoperatively, patients with low BMI should undergo systematic nutritional assessment and, if indicated, receive dietary counseling or supplementation to optimize body composition prior to surgery. Intraoperatively, surgeons should exercise meticulous technique during cement injection—such as using higher-viscosity cement, controlled injection volume, and real-time imaging—to minimize the risk of intradiscal leakage, which was strongly associated with refracture in our cohort. Postoperatively, low HU values on CT can serve as an accessible and reliable proxy for poor bone quality, highlighting the need for intensified anti-osteoporosis pharmacotherapy (e.g., bisphosphonates, anabolic agents) alongside fall prevention and lifestyle modification. Integrating these factors into a structured risk-stratification protocol may enable more personalized management, potentially reducing the incidence of SVF and improving long-term outcomes in elderly OVCF patients undergoing PVP.

This study has certain limitations. First, the sample size is relatively small, which may introduce selection bias. Second, this is a single-center study, making it difficult to generalize the findings across the Chinese population. Third, data on long-term corticosteroid use—a known risk factor for secondary osteoporosis and fracture—were not available in this retrospective cohort, which may have introduced unmeasured confounding. Finally, this study cannot establish a causal relationship between the identified risk factors and vertebral refracture. Prospective, multicenter, randomized large-sample studies should be conducted to further investigate the risk factors for refracture after PVP for the treatment of OVCF.

## Conclusions

In conclusion, BMI, intradiscal cement leakage, and HU values are identified as risk factors for SVF after PVP for the treatment of OVCF. Patients with low BMI, intradiscal cement leakage, and low HU values are at higher risk of refracture. Measures should be taken to mitigate or even prevent the occurrence of refracture following PVP for OVCF, thereby alleviating patient suffering and reducing the clinical and healthcare burden.

## Data Availability

The raw data supporting the conclusions of this article will be made available by the authors, without undue reservation.
